# The therapeutic potential of endogenous hippocampal stem cells for the treatment of neurological disorders

**DOI:** 10.3389/fncel.2013.00005

**Published:** 2013-01-28

**Authors:** Chanel J. Taylor, Dhanisha J. Jhaveri, Perry F. Bartlett

**Affiliations:** Queensland Brain Institute, The University of QueenslandBrisbane, QLD, Australia

**Keywords:** hippocampus, neural stem cell, neural precursor cell, stem cell therapy, endogenous adult neurogenesis

## Abstract

While it is now well-established that resident populations of stem and progenitor cells drive neurogenesis in the adult brain, a growing body of evidence indicates that these new neurons play a pivotal role in spatial learning, memory, and mood regulation. As such, interest is gathering to develop strategies to harness the brain's endogenous reservoir of stem and progenitor cells, with the view that newborn neurons may help overcome the loss of neural and cognitive function that occurs during neurodegenerative disease and psychiatric illness. Here we review evidence for the presence of endogenous stem cell populations in the adult hippocampus, especially large pools of latent stem and precursor cells, and the ways in which these populations can be stimulated to produce new neurons. While the translation of this research from animal models to human application is still in its infancy, understanding in detail the cellular and molecular mechanisms that regulate endogenous neurogenesis, offers the potential to use this innate reservoir of precursors to produce neurons that may be able to mitigate against cognitive decline and mood disorders.

## Introduction

Stem cells have the remarkable ability to self-renew, divide and differentiate into diverse cell types. We are starting to realize the vast implications embryonic and adult stem cells may have for regenerative medicine. In particular, there is a great degree of interest in the development of strategies to harness the therapeutic potential of endogenous neural stem cells in the adult brain for their use in the treatment of neurological disorders. With precise and controlled manipulation, stem cells may have the capacity to replace damaged neurons, halt the progression of neurodegenerative diseases, and ultimately recover function in damaged area(s) of the brain. Since 1992, it has been know that endogenous stem and precursor cells reside in the brains of adult animals (Reynolds and Weiss, 1992; Richards et al., 1992), primarily, though by no means exclusively, in two major areas of the adult brain—the subgranular zone (SGZ) of the hippocampus, and the subventricular zone of the lateral ventricles—and have been shown to continuously generate functional new neurons throughout adulthood. Furthermore, considering recent studies demonstrating the crucial role of newborn neurons in learning and memory formation (Deng et al., 2010; Marin-Burgin and Schinder, 2012) and mood regulation (Sahay and Hen, 2007), the enhancement of hippocampal neurogenesis has immense potential for the treatment of age-associated cognitive decline, dementia, and mental health disorders.

Adult hippocampal neurogenesis in the SGZ is a tightly orchestrated process, involving the maintenance, activation and proliferation of the stem cells; differentiation and migration of the intermediate progenitors; and maturation of the newborn neurons (Bergami and Berninger, 2012; Jhaveri et al., 2012; Lugert et al., 2012). Following successful integration into the neuronal network, these neurons are able to contribute to hippocampal function, as well as sculpt the existing circuitry (Song et al., 2012). A host of intrinsic and extrinsic factors regulates each of these developmental stages (Zhao et al., 2008; Ming and Song, 2011; Jhaveri et al., 2012). To exploit the brain's endogenous reservoir of hippocampal neural stem cells as a potential therapeutic strategy, it is necessary to examine (1) the properties of stem cells that enable them to proliferate, and (2) the methods that can be employed to stimulate their proliferation and neuronal differentiation.

## The quiescent population of stem and progenitor cells in the adult hippocampus

As mentioned, a *bona fide* stem cell must have the defining characteristics of self-renewal through mitotic division, and the ability to differentiate into different cell types. On the other hand, if a cell has limited self-renewal and restricted lineage potential, it is classified as a progenitor cell (Bonaguidi et al., 2011). Without specific techniques to distinguish between these two cell types, stem and progenitor cells are often collectively referred to as precursor cells. Nevertheless, with our advancing understanding of the transcription factors and markers expressed during the different stages of neurogenesis (Hodge and Hevner, 2011; von Bohlen und Halbach, 2011), the introduction of constitutive reporters and inducible transgenic mouse lines (Dhaliwal and Lagace, 2011), and morphological criteria, successful identification of stem and progenitor cells is becoming somewhat easier.

Nevertheless, because unequivocal identification of the potential of a single precursor *in vivo* is still very difficult, characterization and enumeration of stem and progenitor cells in the hippocampus has largely arisen from *in vitro* studies utilizing the neurosphere assay (Bull and Bartlett, 2005; Walker et al., 2008; Jhaveri et al., 2010). When dissociated precursor cells from adult hippocampal tissue are exposed to mitogens, such as epidermal growth factor (EGF) and basic fibroblast growth factor (bFGF), they proliferate to form a ball of cells called a neurosphere. We have found the size of a neurosphere reflects the proliferative capacity of the original precursor cell; for instance, a stem cell with great proliferative and self-renewal potential can generate a neurosphere over 250 μm in diameter, whereas a progenitor cell with limited self-replication generates a neurosphere ~50 μm in diameter (Walker et al., 2008; Jhaveri et al., 2010). Using this *in vitro* approach, our laboratory was the first to report the presence of a large and previously unidentified population of predominantly quiescent stem cells in the adult mouse hippocampus (Walker et al., 2008). In this study, dissociated hippocampal cells were cultured with depolarizing levels of potassium chloride (KCl), which activated a quiescent population of precursor cells to proliferate and produce a more than two-fold increase in number of neurospheres compared to control conditions. Furthermore, a small number (approximately eight per hippocampus) of very large neurospheres (>250 μm in diameter) were obtained, which displayed the stem cell properties of self-renewal and multipotentiality. When the active and rapidly dividing precursor population (i.e., precursors that formed neurospheres under non-depolarizing conditions) were ablated with the anti-mitotic agent AraC (cytosine-b-D-arabinofuranoside), the quiescent pool of precursor cells remained intact, and responded to high KCl stimulation (Walker et al., 2008). Recently, we have shown that stimulation with the monoaminergic neurotransmitter, norepinephrine, directly activated another population of latent stem and precursor cells, via β3-adrenergic receptors, in the adult hippocampus, which is quite distinct from the KCl-activated population (Jhaveri et al., 2010). This raises the tantalizing prospect that these distinct populations may have give rise to subtly different populations of neuronal progeny. Importantly, a recent *in vivo* clonal analysis, using sparsely labeled and inducible Nestin-positive cells, confirmed the existence of a small population—of similar size to that we identified *in vitro—of quiescent* stem cells in the hippocampus that are also capable of self-renewal and multipotent differentiation (Bonaguidi et al., 2011).

The existence of the quiescent stem cell population in the hippocampus has important ramifications for the use of neurogenesis as a therapeutic strategy for functional recovery following brain injury and disease. Given that stem cells in the quiescent pool are usually dormant, and have a seemingly limitless capacity for proliferation, this population offers an untapped resource for the activation and augmentation of neurogenesis. Thus, the most direct and robust method of increasing the rate of neurogenesis will be to activate the quiescent stem cell pool; however, unless self-renewal in the quiescent population is precisely maintained by a number of factors, including Notch signaling (Pierfelice et al., 2011), its reservoir of stem cells may become depleted, leading to the premature loss of neurogenesis. Furthermore, the mechanisms regulating neuronal differentiation, survival and integration must also be well-managed to obtain successful neurogenesis.

**Figure 1:**
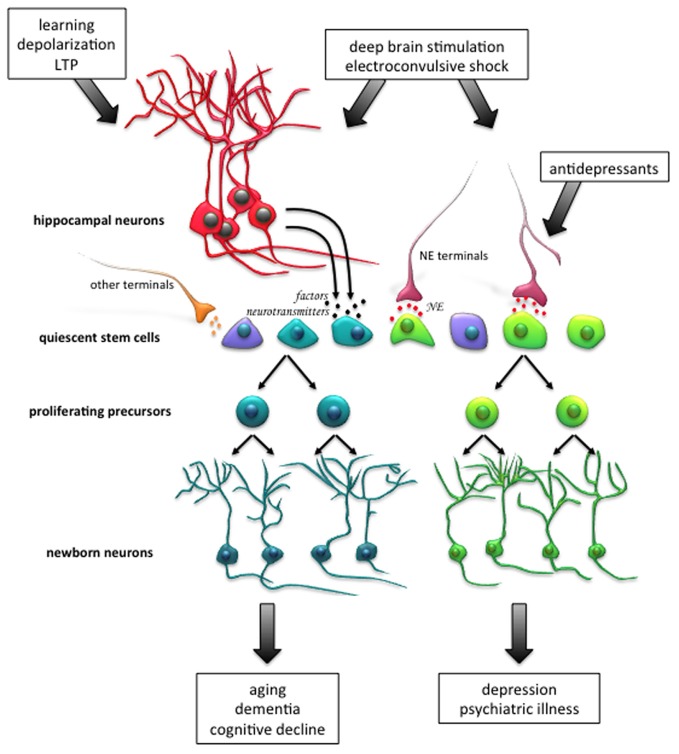
**Activation of the endogenous population of quiescent stem cells in the adult hippocampus as a therapeutic strategy for neural injury and disease.** Enhancement of neurogenesis, through activation of the quiescent stem cell pool, may facilitate neuronal repair and recovery of cognitive function following aging, neuronal disorders, psychiatric conditions, and neurodegenerative diseases. For instance, neuronal activity, such as that which occurs during learning, the induction of long-term potentiation (LTP) and *in vitro* depolarization, potently activates quiescent stem cell proliferation. A different subpopulation of stem cells is activated following exposure to norepinephrine (NE), and it is thought that this population responds to NE-mediated antidepressant treatment which can alleviate clinical depression. Patterns of electrical stimulation, such as deep brain stimulation and electroconvulsive shock, which are currently used to treat many neurodegenerative and psychiatric conditions in human patients, have also been shown to promote proliferation in animal models. Newborn neurons arising from the distinct progenitor cell pools may have differential roles in the functional outcome of neurodegenerative disease and psychiatric illness. Abbreviations: NE, norepinephrine; LTP, long-term potentiation.

In terms of origin, there may exist a reciprocal relationship between the quiescent and active stem cells in the hippocampus, in that they are able to give rise to one another (Suh et al., 2007; Lavado et al., 2010). Nevertheless, while these two subpopulations are kept in a delicate equilibrium by a number of factors, the precise mechanisms underlying how these pools are regulated and maintained, and the flux between their quiescent and active states, requires further characterization. Nonetheless, we believe that molecular signals may “prime” quiescent precursor cells to be able to respond to proliferative signals coming from the hippocampal niche (see Figure 1). Whether these signals come from newborn neurons, mature granule cells or surrounding glial cells as a result of neuronal activity is just beginning to be understood.

## Activation of the quiescent stem cell population

The SGZ of the hippocampus is enriched with a vast number of projections arising from a number of areas throughout the brain, which modulate neuronal activity in the dentate gyrus. Neurotransmitters released from these projections have been shown to affect multiple stages of hippocampal neurogenesis (Vaidya et al., 2007). Furthermore, growth factors and signaling molecules that are upregulated following high-intensity neuronal activity may also modulate hippocampal neurogenesis (e.g., Balkowiec and Katz, 2002; Chen et al., 2006). For instance, environmental novelty and neuronal activity-mediated activation of noradrenergic terminals in the dentate gyrus have been shown to result in the release of norepinephrine (Harley, 2007; King and Williams, 2009; Neugebauer et al., 2009), which in turn has been shown to directly activate the Hes5-expressing quiescent pool of stem and precursor cells in the adult hippocampus (Jhaveri et al., 2010). While there is a burgeoning number of putative candidates that regulate neurogenesis, we focus here on the role of neuronal activity in the activation of the quiescent stem cell pool in the adult hippocampus.

### Electrophysiological stimulation

Learning-related electrophysiological stimulation of the hippocampus has been widely shown to promote precursor cell proliferation and the survival of newborn neurons in the SGZ (Bruel-Jungerman et al., 2006; Chun et al., 2006; Toda et al., 2008; Kitamura et al., 2010; Stone et al., 2011; Kameda et al., 2012). Nevertheless, the mechanisms underlying how the precursor population responds to the induction of LTP have not yet been definitively revealed. We have shown recently that activation of the precursor cell population occurs only following the successful induction of LTP in the dentate gyrus *in vivo* (Kameda et al., 2012). These precursors appear to be the same population that responds to KCl stimulation *in vitro*, as high KCl did not further increase the number of neurospheres in the LTP-induced hippocampal cells. The LTP-mediated activation of precursor cells, and the increase in the production of doublecortin (DCX)-positive neurons observed *in vivo*, was specific to the induction of NMDA-dependent late-LTP, as this effect was blocked by the NMDA receptor antagonist CPP [(6)-3-(2-carboxypiperazin-4-yl)propyl-1-phosphonic acid], administered shortly before the induction of LTP. Similarly, protocols that delivered low-frequency stimulation, or high-frequency stimulation that failed to induce LTP or only induced early-LTP, did not affect precursor activation or proliferation. Interestingly, the magnitude of LTP strongly correlated with the extent of precursor cell activation, suggesting a dose-dependent-like mechanism underlies this effect. While speculative, it is possible that LTP induction somehow primes hippocampal precursor cells to respond to factors that directly activate proliferation. This effect may be temporally transient, given that when the hippocampus was dissociated 4 days following the induction of LTP, no significant increase in activation of precursor cells was seen *in vitro* (Kameda et al., 2012). Nonetheless, these observations are the first to provide direct evidence that patterns of synaptic activity associated with learning and memory can activate latent precursor cells in the adult hippocampus, as well as increase neuron production. More studies are required to investigate the mechanism of precursor cell activation following defined patterns of physiological stimulation, considering that synaptic plasticity, learning, and neurogenesis are intimately linked.

Other patterns of electrical stimulation have also been shown promote neurogenesis. Stimulation protocols used to induce electroconvulsive seizure (ECS) robustly increase the proliferation and survival of progenitor cells in rodents (Madsen et al., 2000; Ito et al., 2010) and non-human primates (Perera et al., 2007). Furthermore, recent clinical advances are starting to show that deep brain stimulation (DBS) of the human brain can successfully treat many psychiatric and neurological conditions (reviewed in Cramer et al., 2011; Moreines et al., 2011; Hamani and Temel, 2012), and has been shown to affect neurogenesis in rodent models of disease (e.g., Khaindrava et al., 2011). While the link between electrical stimulation of the human brain and neurogenesis awaits investigation, a phase I clinical trial assessing the effect of DBS targeting the fornix/hypothalamus in patients experiencing mild Alzheimer's disease has revealed that some patients showed a slower rate of cognitive decline over a 12-month period (Laxton et al., 2010). Therefore, given the ability of electrical stimulation to enhance endogenous neurogenesis, and the strong links between neurogenesis in the SGZ and improvement in cognitive function (e.g., Deng et al., 2010; Marin-Burgin and Schinder, 2012), stimulating the quiescent population of stem and precursor cells to augment endogenous neurogenesis to recover or enhance cognitive function in neurodegenerative disease in an alluring prospect. Nevertheless, the identity of the latent precursor pool(s) that respond to the different patterns of electrical activity (e.g., LTP-inducing high-frequency stimulation, ECS and DBS) to generate new neurons remains to be addressed.

## Hippocampal neurogenesis and aging

It is now well-established that hippocampal neurogenesis dramatically decreases with age. Aged animals show significantly less precursor and progenitor cell proliferation, neuronal differentiation and newborn neuron survival (reviewed by Lee et al., 2012). This is also true for the number of neurospheres that can be generated from mice as they age, which by 18 months in the hippocampus, declines by about 80% (Walker et al., 2008). The cause of this neurogenic decline over time is not well-understood, as many factors underlying the maintenance and regulation of neurogenesis are altered in the aged brain (Artegiani and Calegari, 2012; Lee et al., 2012). Nonetheless, given the negative impact of aging on hippocampal neurogenesis (Lee et al., 2012) and cognitive function (e.g., Rosenzweig and Barnes, 2003), stimulating the endogenous stem cell pool in the aged brain may be able to restore the memory and cognitive deficits observed in age-associated and mild cognitive impairment, and neurodegenerative diseases, such as Alzheimer's disease (e.g., Laxton et al., 2010).

To develop an effective strategy to enhance neurogenesis in the aged hippocampus, it is important to identify whether the age-related reduction in proliferation and neurogenesis results from an increase in stem cell quiescence over time (Lugert et al., 2010; Bonaguidi et al., 2011), or depletion of the stem cell pool (Encinas et al., 2011). A study from our laboratory has shown that the aged brain does in fact retain a population of quiescent stem cells, which have the capacity to respond to stimuli that activate proliferation *in vitro* (Walker et al., 2008). Indeed, physical exercise, which strongly increases stem cell proliferation and neuron production (van Praag et al., 1999; Kronenberg et al., 2003; Lugert et al., 2010), has been positively correlated with improved hippocampal-dependent cognitive performance in aged animals (van Praag et al., 2005; Marlatt et al., 2012). While a number of factors may underlie the neurogenic and cognitive restoration observed in aged animals following physical exercise (see Lee et al., 2012), a strong case has recently been made for the involvement of growth hormone in this process in the subventricular zone (SVZ) (Blackmore et al., 2009, 2012). Further investigation is required to show whether growth hormone mediates exercise-induced activation of the quiescent precursor cell population in the aged hippocampus.

We have shown recently that microglia are also very important in regulating the activation of precursors in the aging animal and after exercise (Vukovic et al., 2012). Removal of microglia from the hippocampal population in aged animals leads to a significant increase in precursor activation, indicating the low number of active precursors may be due to the inhibitory effects of microglia, probably through the release of cytokines. Conversely, microglia removed from hippocampal cultures of young animals has no effect, but their removal following running decreases neurosphere numbers, indicating microglia play a positive role in the activation process. We have shown that the ability of microglia to increase precursor activity is due to the release of the chemokine, CX3C (also known as fractalkine) from neurons, and that levels of this ligand decrease dramatically with age (Vukovic et al., 2012). These results open the possibility that raising CX3C levels may increase neurogenesis in the aging animal. In addition, we have found that interferon gamma, another cytokine found normally in the hippocampus that is raised during inflammatory conditions, is also a potent inhibitor of precursor activation. Interestingly, this inhibition can be partially blocked by factors released from microglia (Li et al., 2010). This environment of negative and positive regulators of hippocampal precursor activation poses some challenges, especially in the aged animal, as we try to stimulate the appropriate neurogenic production in the hippocampus in a way that leads to the enhancement of function in the aged brain suffering cognitive decline, as outlined in Figure 1.

## Hippocampal neurogenesis in mood-related disorders

Another area of research in which hippocampal neurogenesis has received considerable attention is in the investigation of animal models of depression, and specifically, the mechanisms underlying the actions of antidepressants (reviewed by Sahay and Hen, 2007). Changes in hippocampal neurogenesis have now been implicated in the etiology of depression; furthermore, current antidepressant treatments are thought to target these neurogenic changes (Santarelli et al., 2003; David et al., 2009; Snyder et al., 2011; Surget et al., 2011). Given the Australian National Survey of Mental Health and Wellbeing (2007) reported that about 20% of the population experience some form of mental disorder within a 12-month period, with mood-related disorders such as anxiety and depression being the most prevalent, a thorough understanding of the cellular and molecular mechanisms underlying these conditions is necessary to be able to identify novel therapeutic strategies.

Studies have shown an enhancement of hippocampal neurogenesis following chronic treatment with clinical antidepressants (Malberg et al., 2000; Duman, 2004; Perera et al., 2007). In particular, ECS, which initiate rapid onset of antidepressant action, have been shown to increase the proliferation of GFAP- and Sox2-positive quiescent stem cells in the SGZ (Segi-Nishida et al., 2008). More recently, we have shown that norepinephrine, and antidepressants that block the reuptake of norepinephrine (such as reboxetine and atomoxetine), also increase proliferation by directly activating the quiescent population of precursors in the hippocampus (Jhaveri et al., 2010). In fact, the neurogenesis-depression hypothesis proposes that the functional effect of antidepressant treatment is mediated by the addition of new neurons to the hippocampal network (Kempermann and Kronenberg, 2003). This hypothesis is supported by the strong correlation that exists between the timeline of new neuron production and the delayed onset of the beneficial effects of antidepressant medication (Nestler et al., 2002; Sahay and Hen, 2007). Importantly, the blockade of hippocampal neurogenesis has been shown to inhibit some, but not all, of the therapeutic-like effects of antidepressants in rodent models (Santarelli et al., 2003; David et al., 2009); however, no anxiety/depressive-like behavior has been observed in animals lacking neurogenesis, suggesting impairment in neurogenesis alone is not sufficient to cause mood-related disorders. To better understand the etiology of depression, a detailed understanding of the interactions between the hippocampus and other brain areas is needed, with an emphasis on the precise molecular mechanisms by which newborn neurons in the hippocampus ameliorate depression.

The hippocampus is known to play an important role in regulating stress through the hypothalamic-pituitary-adrenal (HPA) axis (Herman et al., 1989; Mizoguchi et al., 2003). While several studies have shown that chronic stress or an elevation of cortisol levels decreases neurogenesis (Gould et al., 1998; Murray et al., 2008; Lagace et al., 2010), two recent studies have suggested that newborn hippocampal neurons mediate the feedback inhibition of the HPA axis following stress, thereby regulating blood cortisol levels (Snyder et al., 2011; Surget et al., 2011). Surget and colleagues (2011) have shown that in animals in which neurogenesis has been ablated, restraint stress leads to a longer-lasting elevation in cortisol levels compared to animals with intact neurogenesis. In addition, neurogenesis-ablated mice show increased food avoidance in a novel environment, indicating higher levels of anxiety. Importantly, Surget and colleagues (2011) have shown that fluoxetine treatment, in the absence of ongoing neurogenesis, fails to restore HPA axis activity and function in neurogenesis-ablated animals subjected to unpredictable chronic stress.

Taken together, it appears that functional hippocampal neurogenesis plays an important role in preventing the onset of mood-related disorders following chronic stress, and importantly, acts as a substrate for the therapeutic action of antidepressants. Enhancing the production of newborn neurons by harnessing the potential of endogenous precursor populations may therefore be an extremely useful therapeutic approach (see Figure 1). Given that at least two large latent precursor populations have been uncovered so far in the hippocampus (Walker et al., 2008; Jhaveri et al., 2010), driving neuronal production by enhancing the proliferative activity could indeed boost hippocampal neurogenesis. While the monoaminergic family of drugs remain the front line choice for the treatment of mood disorders, their ineffectiveness and distressing side effects warrants the discovery of new antidepressant treatments (Ellis, 2004). More effective, rapid-acting and long-lasting antidepressants may emerge from undertaking a detailed molecular characterization of these latent precursor pools, as well their neuronal progeny, which may lead to the identification of novel targets for drug development (Jhaveri et al., 2012). Finally, enhancing neuronal production may also prove beneficial for neurodegenerative diseases that involve significant cellular and functional loss in the hippocampus.

## Conclusions

Resident populations of endogenous stem and progenitor cells drive the production of new neurons in the adult hippocampus. These precursor pools are carefully regulated and precisely maintained, such that upon pathological insult or homeostatic perturbations, proliferation can be rapidly enhanced to replace neurons lost due to damage or dysfunction. Nevertheless, enhancing proliferation alone is often not enough to generate sufficient numbers of new neurons and restore normal function. As such, various methods of further activating and amplifying stem and progenitor cells in the hippocampus have provided some positive results for the recovery of hippocampal-dependent behavioral functions in animal models.

While we have discussed the stimulation of endogenous neural stem cells in a number of disease states in rodent models, effective therapies for human application must be tailored to the endogenous microenvironment of the human neurogenic niche. Currently, there are limited studies providing evidence for ongoing hippocampal neurogenesis in the adult human brain (Eriksson et al., 1998; Blumcke et al., 2001; Sanai et al., 2004); furthermore, the cell cycle length in primates appears to be substantially longer than in rodents, indicating a much slower rate of proliferation and precursor cell turnover (Breunig et al., 2011; Curtis et al., 2012). Additional research is therefore required to examine the extent of adult neurogenesis in the human brain, for instance, using post-mortem brain tissue from age-matched controls, as well as patients suffering from mood and neurological disorders. Given that post-mortem brain tissue can only provide a temporal snapshot of neurogenesis, advances in live imaging techniques that can track cell proliferation in the human hippocampus at high resolution will open an exciting avenue for future investigation. In addition, the development of sophisticated drug delivery technology is needed to specifically target and activate proliferation of the endogenous populations of stem cells, and to facilitate the differentiation, migration, integration, and survival of newborn neurons. Nevertheless, a detailed understanding of the fundamental mechanisms regulating neurogenesis in animal models will be crucial in order to utilize the endogenous stem cell populations as a therapeutic approach in patients suffering from brain injury and disease.

### Conflict of interest statement

The authors declare that the research was conducted in the absence of any commercial or financial relationships that could be construed as a potential conflict of interest.

## References

[B1] Artegiani B. Calegari F. (2012). Age-related cognitive decline: can neural stem cells help us? Aging 4, 176–186 22466406 10.18632/aging.100446 PMC3348478

[B2] Australian Bureau of Statistics. (2007). National Survey of Mental Health and Wellbeing: Summary of Results. Canberra, ACT: ABS

[B3] Balkowiec A. Katz D. M. (2002). Cellular mechanisms regulating activity-dependent release of native brain-derived neurotrophic factor from hippocampal neurons. J. Neurosci. 22, 10399–10407 12451139 10.1523/JNEUROSCI.22-23-10399.2002 PMC6758764

[B4] Bergami M. Berninger B. (2012). A fight for survival: the challenges faced by a newborn neuron integrating in the adult hippocampus. Dev. Neurobiol. 72, 1016–1031 10.1002/dneu.22025 22488787

[B5] Blackmore D. G. Golmohammadi M. G. Large B. Waters M. J. Rietze R. L. (2009). Exercise increases neural stem cell number in a growth hormone-dependent manner, augmenting the regenerative response in aged mice. Stem Cells 27, 2044–2052 10.1002/stem.120 19544415

[B6] Blackmore D. G. Reynolds B. A. Golmohammadi M. G. Large B. Aguilar R. M. Haro L. (2012). Growth hormone responsive neural precursor cells reside within the adult mammalian brain. Sci. Rep. 2:250 10.1038/srep00250 22355762 PMC3274722

[B7] Blumcke I. Schewe J. C. Normann S. Brustle O. Schramm J. Elger C. E. (2001). Increase of nestin-immunoreactive neural precursor cells in the dentate gyrus of pediatric patients with early-onset temporal lobe epilepsy. Hippocampus 11, 311–321 10.1002/hipo.1045 11769312

[B8] Bonaguidi M. A. Wheeler M. A. Shapiro J. S. Stadel R. P. Sun G. J. Ming G. L. (2011). *In vivo* clonal analysis reveals self-renewing and multipotent adult neural stem cell characteristics. Cell 145, 1142–1155 10.1016/j.cell.2011.05.024 21664664 PMC3124562

[B9] Breunig J. J. Haydar T. F. Rakic P. (2011). Neural stem cells: historical perspective and future prospects. Neuron 70, 614–625 10.1016/j.neuron.2011.05.005 21609820 PMC3225274

[B10] Bruel-Jungerman E. Davis S. Rampon C. Laroche S. (2006). Long-term potentiation enhances neurogenesis in the adult dentate gyrus. J. Neurosci. 26, 5888–5893 10.1523/JNEUROSCI.0782-06.2006 16738230 PMC6675234

[B11] Bull N. D. Bartlett P. F. (2005). The adult mouse hippocampal progenitor is neurogenic but not a stem cell. J. Neurosci. 25, 10815–10821 10.1523/JNEUROSCI.3249-05.2005 16306394 PMC6725873

[B12] Chen J. Y. Park C. S. Tang S. J. (2006). Activity-dependent synaptic Wnt release regulates hippocampal long term potentiation. J. Biol. Chem. 281, 11910–11916 10.1074/jbc.M511920200 16501258

[B13] Chun S. K. Sun W. Park J. J. Jung M. W. (2006). Enhanced proliferation of progenitor cells following long-term potentiation induction in the rat dentate gyrus. Neurobiol. Learn. Mem. 86, 322–329 10.1016/j.nlm.2006.05.005 16824772

[B14] Cramer S. C. Sur M. Dobkin B. H. O'Brien C. Sanger T. D. Trojanowski J. Q. (2011). Harnessing neuroplasticity for clinical applications. Brain 134, 1591–1609 10.1093/brain/awr039 21482550 PMC3102236

[B15] Curtis M. A. Low V. F. Faull R. L. M. (2012). Neurogenesis and progenitor cells in the adult human brain: a comparison between hippocampal and subventricular progenitor proliferation. Dev. Neurobiol. 72, 990–1005 10.1002/dneu.22028 22539366

[B16] David D. J. Samuels B. A. Rainer Q. Wang J. W. Marsteller D. Mendez I. (2009). Neurogenesis-dependent and -independent effects of fluoxetine in an animal model of anxiety/depression. Neuron 62, 479–493 10.1016/j.neuron.2009.04.017 19477151 PMC2759281

[B17] Deng W. Aimone J. B. Gage F. H. (2010). New neurons and new memories: how does adult hippocampal neurogenesis affect learning and memory? Nat. Rev. Neurosci. 11, 339–350 10.1038/nrn2822 20354534 PMC2886712

[B18] Dhaliwal J. Lagace D. C. (2011). Visualization and genetic manipulation of adult neurogenesis using transgenic mice. Eur. J. Neurosci. 33, 1025–1036 10.1111/j.1460-9568.2011.07600.x 21395845

[B19] Duman R. S. (2004). Depression: a case of neuronal life and death? Biol. Psychiatry 56, 140–145 10.1016/j.biopsych.2004.02.033 15271581

[B20] Ellis P. (2004). Australian and New Zealand clinical practice guidelines for the treatment of depression. Aust. N.Z. J. Psychiatry 38, 389–407 10.1111/j.1440-1614.2004.01377.x 15209830

[B21] Encinas J. M. Michurina T. V. Peunova N. Park J. H. Tordo J. Peterson D. A. (2011). Division-coupled astrocytic differentiation and age-related depletion of neural stem cells in the adult hippocampus. Cell Stem Cell 8, 566–579 10.1016/j.stem.2011.03.010 21549330 PMC3286186

[B23] Eriksson P. S. Perfilieva E. Bjork-Eriksson T. Alborn A. M. Nordborg C. Peterson D. A. (1998). Neurogenesis in the adult human hippocampus. Nat. Med. 4, 1313–1317 10.1002/dneu.22028 9809557

[B24] Gould E. Tanapat P. McEwen B. S. Flugge G. Fuchs E. (1998). Proliferation of granule cell precursors in the dentate gyrus of adult monkeys is diminished by stress. Proc. Natl. Acad. Sci. U.S.A. 95, 3168–3171 9501234 10.1073/pnas.95.6.3168 PMC19713

[B25] Hamani C. Temel Y. (2012). Deep brain stimulation for psychiatric disease: contributions and validity of animal models. Sci. Transl. Med. 4:142rv8 10.1126/scitranslmed.3003722 22786683

[B26] Harley C. W. (2007). Norepinephrine and the dentate gyrus: a comphrehensive guide to structure, function, and clinical implications. Prog. Brain Res. 163, 299–318 10.1016/S0079-6123(07)63018-0 17765726

[B27] Herman J. P. Schafer M. K. H. Young E. A. Thompson R. Douglass J. Akil H. (1989). Evidence for hippocampal regulation of neuroendocrine neurons of the hypothalamo-pituitary-adrenocortical axis. J. Neurosci. 9, 3072–3082 2795152 10.1523/JNEUROSCI.09-09-03072.1989 PMC6569679

[B28] Hodge R. D. Hevner R. F. (2011). Expression and actions of transcription factors in adult hippocampal neurogenesis. Dev. Neurobiol. 71, 680–689 10.1002/dneu.20882 21412988 PMC3134120

[B29] Ito M. Seki T. Liu J. A. Nakamura K. Namba T. Matsubara Y. (2010). Effects of repeated electroconvulsive seizure on cell proliferation in the rat hippocampus. Synapse 64, 814–821 10.1002/syn.20796 20340175

[B30] Jhaveri D. J. Mackay E. W. Hamlin A. S. Marathe S. V. Nandam L. S. Vaidya V. A. (2010). Norepinephrine directly activates adult hippocampal precursors via beta(3)-adrenergic receptors. J. Neurosci. 30, 2795–2806 10.1523/JNEUROSCI.3780-09.2010 20164362 PMC2837927

[B31] Jhaveri D. J. Taylor C. J. Bartlett P. F. (2012). Activation of different neural precursor populations in the adult hippocampus: does this lead to new neurons with discrete functions? Dev. Neurobiol. 72, 1044–1058 10.1002/dneu.22027 22505142

[B32] Kameda M. Taylor C. J. Walker T. L. Black D. M. Abraham W. C. Bartlett P. F. (2012). Activation of latent precursors in the hippocampus is dependent on long-term potentiation. Transl. Psychiatry 2:e72 10.1038/tp.2011.70 22832734 PMC3309542

[B33] Kempermann G. Kronenberg G. (2003). Depressed new neurons– adult hippocampal neurogenesis and a cellular plasticity hypothesis of major depression. Biol. Psychiatry 54, 499–503 10.1016/S0006-3223(03)00319-6 12946878

[B34] Khaindrava V. Salin P. Melon C. Ugrumov M. Kerkerian-Le-Goff L. Daszuta A. (2011). High frequency stimulation of the subthalamic nucleus impacts adult neurogenesis in a rat model of Parkinson's disease. Neurobiol. Dis. 42, 284–291 10.1016/j.nbd.2011.01.018 21296669

[B35] King S. O. 2nd. Williams C. L. (2009). Novelty-induced arousal enhances memory for cued classical fear conditioning: interactions between peripheral adrenergic and brainstem glutamatergic systems. Learn. Mem. 16, 625–634 10.1101/lm.1513109 19794188

[B36] Kitamura T. Saitoh Y. Murayama A. Sugiyama H. Inokuchi K. (2010). LTP induction within a narrow critical period of immature stages enhances the survival of newly generated neurons in the adult rat dentate gyrus. Mol. Brain 3, 3–13 10.1186/1756-6606-3-13 20426820 PMC2868842

[B37] Kronenberg G. Reuter K. Steiner B. Brandt M. D. Jessberger S. Yamaguchi M. (2003). Subpopulations of proliferating cells of the adult hippocampus respond differently to physiologic neurogenic stimuli. J. Comp. Neurol. 467, 455–463 10.1002/cne.10945 14624480

[B38] Lagace D. C. Donovan M. H. DeCarolis N. A. Farnbauch L. A. Malhotra S. Berton O. (2010). Adult hippocampal neurogenesis is functionally important for stress-induced social avoidance. Proc. Natl. Acad. Sci. U.S.A. 107, 4436–4441 10.1073/pnas.0910072107 20176946 PMC2840117

[B39] Lavado A. Lagutin O. V. Chow L. M. L. Baker S. J. Oliver G. (2010). Prox1 is required for granule cell maturation and intermediate progenitor maintenance during brain neurogenesis. PLoS Biol. 8:e1000460 10.1371/journal.pbio.1000460 20808958 PMC2923090

[B40] Laxton A. W. Tang-Wai D. F. McAndrews M. P. Zumsteg D. Wennberg R. Keren R. (2010). A phase I trial of deep brain stimulation of memory circuits in Alzheimer's disease. Ann. Neurol. 68, 521–534 10.1002/ana.22089 20687206

[B41] Lee S. W. Clemenson G. D. Gage F. H. (2012). New neurons in an aged brain. Behav. Brain Res. 227, 497–507 10.1016/j.bbr.2011.10.009 22024433 PMC3264739

[B42] Li L. Walker T. L. Zhang Y. Mackay E. W. Bartlett P. F. (2010). Endogenous interferon gamma directly regulates neural precursors in the non-inflammatory brain. J. Neurosci. 30, 9038–9050 10.1523/JNEUROSCI.5691-09.2010 20610738 PMC6632462

[B43] Lugert S. Basak O. Knuckles P. Haussler U. Fabel K. Gotz M. (2010). Quiescent and active hippocampal neural stem cells with distinct morphologies respond selectively to physiological and pathological stimuli and aging. Cell Stem Cell 6, 445–456 10.1016/j.stem.2010.03.017 20452319

[B44] Lugert S. Vogt M. Tchorz J. S. Muller M. Giachino C. Taylor V. (2012). Homeostatic neurogenesis in the adult hippocampus does not involve amplification of Ascl1(high) intermediate progenitors. Nat. Commun. 3:670 10.1038/ncomms1670 22334073

[B45] Madsen T. M. Treschow A. Bengzon J. Bolwig T. G. Lindvall O. Tingstrom A. (2000). Increased neurogenesis in a model of electroconvulsive therapy. Biol. Psychiatry 47, 1043–1049 10862803 10.1016/s0006-3223(00)00228-6

[B46] Malberg J. E. Eisch A. J. Nestler E. J. Duman R. S. (2000). Chronic antidepressant treatment increases neurogenesis in adult rat hippocampus. J. Neurosci. 20, 9104–9110 11124987 10.1523/JNEUROSCI.20-24-09104.2000 PMC6773038

[B47] Marin-Burgin A. Schinder A. F. (2012). Requirement of adult-born neurons for hippocampus-dependent learning. Behav. Brain Res. 227, 391–399 10.1016/j.bbr.2011.07.001 21763727

[B48] Marlatt M. W. Potter M. C. Lucassen P. J. van Praag H. (2012). Running throughout middle-age improves memory function, hippocampal neurogenesis, and BDNF levels in female C57BL/6J mice. Dev. Neurobiol. 72, 943–952 10.1002/dneu.22009 22252978 PMC3485396

[B49] Ming G. L. Song H. (2011). Adult neurogenesis in the mammalian brain: significant answers and significant questions. Neuron 70, 687–702 10.1016/j.neuron.2011.05.001 21609825 PMC3106107

[B50] Mizoguchi K. Ishige A. Aburada M. Tabira T. (2003). Chronic stress attenuates glucocorticoid negative feedback: involvement of the prefrontal cortex and hippocampus. Neuroscience 119, 887–897 10.1016/S0306-4522(03)00105-2 12809708

[B51] Moreines J. L. McClintock S. M. Holtzheimer P. E. (2011). Neuropsychologic effects of neuromodulation techniques for treatment-resistant depression: a review. Brain Stimul. 4, 17–27 10.1016/j.brs.2010.01.005 21255751 PMC3023999

[B52] Murray F. Smith D. W. Hutson P. H. (2008). Chronic low dose corticosterone exposure decreased hippocampal cell proliferation, volume and induced anxiety and depression like behaviours in mice. Eur. J. Pharmacol. 583, 115–127 10.1016/j.ejphar.2008.01.014 18289522

[B53] Nestler E. J. Barrot M. DiLeone R. J. Eisch A. J. Gold S. J. Monteggia L. M. (2002). Neurobiology of depression. Neuron 34, 13–25 11931738 10.1016/s0896-6273(02)00653-0

[B54] Neugebauer F. Korz V. Frey J. U. (2009). Modulation of extracellular monoamine transmitter concentrations in the hippocampus after weak and strong tetanization of the perforant path in freely moving rats. Brain Res. 1273, 29–38 10.1016/j.brainres.2009.03.055 19345680

[B55] Perera T. D. Coplan J. D. Lisanby S. H. Lipira C. M. Arif M. Carpio C. (2007). Antidepressant-induced neurogenesis induced neurogenesis in the hippocampus of adult nonhuman primates. J. Neurosci. 27, 4894–4901 10.1523/JNEUROSCI.0237-07.2007 17475797 PMC6672102

[B56] Pierfelice T. Alberi L. Gaiano N. (2011). Notch in the vertebrate nervous system: an old dog with new tricks. Neuron 69, 840–855 10.1016/j.neuron.2011.02.031 21382546

[B57] Reynolds B. A. Weiss S. (1992). Generation of neurons and astrocytes from isolated cells of the adult mammalian central nervous system. Science 255, 1707–1710 10.1126/science.1553558 1553558

[B58] Richards L. J. Kilpatrick T. J. Bartlett P. F. (1992). *De novo* generation of neuronal cells from the adult mouse brain. Proc. Natl. Acad. Sci. U.S.A. 89, 8591–8595 10.1073/pnas.89.18.8591 1528866 PMC49966

[B59] Rosenzweig E. S. Barnes C. A. (2003). Impact of aging on hippocampal function: plasticity, network dynamics, and cognition. Prog. Neurobiol. 69, 143–179 10.1016/S0301-0082(02)00126-0 12758108

[B60] Sahay A. Hen R. (2007). Adult hippocampal neurogenesis in depression. Nat. Neurosci. 10, 1110–1115 10.1038/nn1969 17726477

[B61] Sanai N. Tramontin A. D. Quinones-Hinojosa A. Barbaro N. M. Gupta N. Kunwar S. (2004). Unique astrocyte ribbon in adult human brain contains neural stem cells but lacks chain migration. Nature 427, 740–744 10.1038/nature02301 14973487

[B62] Santarelli L. Saxe M. Gross C. Surget A. Battaglia F. Dulawa S. (2003). Requirement of hippocampal neurogenesis for the behavioral effects of antidepressants. Science 301, 805–809 10.1126/science.1083328 12907793

[B63] Segi-Nishida E. Warner-Schmidt J. L. Duman R. S. (2008). Electroconvulsive seizure and VEGF increase the proliferation of neural stem-like cells in rat hippocampus. Proc. Natl. Acad. Sci. U.S.A. 105, 11352–11357 10.1073/pnas.0710858105 18682560 PMC2516270

[B64] Snyder J. S. Soumier A. Brewer M. Pickel J. Cameron H. A. (2011). Adult hippocampal neurogenesis buffers stress responses and depressive behaviour. Nature 476, 458–461 10.1038/nature10287 21814201 PMC3162077

[B65] Song J. Christian K. M. Ming G. L. Song H. J. (2012). Modification of hippocampal circuitry by adult neurogenesis. Dev. Neurobiol. 72, 1032–1043 10.1002/dneu.22014 22354697 PMC3710549

[B66] Stone S. S. D. Teixeira C. M. DeVito L. M. Zaslavsky K. Josselyn S. A. Lozano A. M. (2011). Stimulation of entorhinal cortex promotes adult neurogenesis and facilitates spatial memory. J. Neurosci. 31, 13469–13484 10.1523/JNEUROSCI.3100-11.2011 21940440 PMC6623309

[B67] Suh H. Consiglio A. Ray J. Sawai T. D'Amour K. A. Gage F. H. (2007). *In vivo* fate analysis reveals the multipotent and self-renewal capacities of Sox2(+) neural stem cells in the adult hippocampus. Cell Stem Cell 1, 515–528 10.1016/j.stem.2007.09.002 18371391 PMC2185820

[B68] Surget A. Tanti A. Leonardo E. D. Laugeray A. Rainer Q. Touma C. (2011). Antidepressants recruit new neurons to improve stress response regulation. Mol. Psychiatry 16, 1177–1188 10.1038/mp.2011.48 21537331 PMC3223314

[B69] Toda H. Hamani C. Fawcett A. P. Hutchison W. D. Lozano A. M. (2008). The regulation of adult rodent hippocampal neurogenesis by deep brain stimulation. J. Neurosurg. 108, 132–138 10.3171/JNS/2008/108/01/0132 18173322

[B70] Vaidya V. A. Vadodaria K. C. Jha S. (2007). Neurotransmitter regulation of adult neurogenesis: putative therapeutic targets. CNS Neurol. Disord. Drug Targets 6, 358–374 18045164 10.2174/187152707783220910

[B71] van Praag H. Kempermann G. Gage F. H. (1999). Running increases cell proliferation and neurogenesis in the adult mouse dentate gyrus. Nat. Neurosci. 2, 266–270 10.1038/6368 10195220

[B72] van Praag H. Shubert T. Zhao C. M. Gage F. H. (2005). Exercise enhances learning and hippocampal neurogenesis in aged mice. J. Neurosci. 25, 8680–8685 10.1523/JNEUROSCI.1731-05.2005 16177036 PMC1360197

[B73] von Bohlen und Halbach O. (2011). Immunohistological markers for proliferative events, gliogenesis, and neurogenesis within the adult hippocampus. Cell Tissue Res. 345, 1–19 10.1007/s00441-011-1196-4 21647561

[B74] Vukovic J. Colditz M. J. Blackmore D. G. Ruitenberg M. J. Bartlett P. F. (2012). Microglia modulate hippocampal neural precursor activity in response to exercise and aging. J. Neurosci. 32, 6435–6443 10.1523/JNEUROSCI.5925-11.2012 22573666 PMC6621117

[B75] Walker T. L. White A. Black D. M. Wallace R. H. Sah P. Bartlett P. F. (2008). Latent stem and progenitor cells in the hippocampus are activated by neural excitation. J. Neurosci. 28, 5240–5247 10.1523/JNEUROSCI.0344-08.2008 18480280 PMC6670644

[B76] Zhao C. Deng W. Gage F. H. (2008). Mechanisms and functional implications of adult neurogenesis. Cell 132, 645–660 10.1016/j.cell.2008.01.033 18295581

